# 

**Published:** 1885-02

**Authors:** 


### CONTENTS:

Art. I—Dental Caries, and its Relations to the Germ Theory of Disease. By G. V. Black, M. D., D. D. S. .... 433

Art. II—Irregularities of the Teeth. By Norman W. Kingsley, D. D. S. .... 452

Art. III—Sale of American Diplomas in Europe. .... 458

Art. IV—The Standard of Work on the Natural Teeth that the Dentist of the Future must be Equal to. .... 463

Art. V—The Utilization of Waste Scraps of Old Amalgams, Etc. By Prof. Chas. Mayr. .... 467

Art. VI—Filling the Permanent Teeth of Children. By Dr. C. M. Marshall .... 469

Illinois State Dental Society .... 471

### EDITORIAL, ETC.

Annual Commencements of The University of Maryland Dental Department .... 472

Some Experiments with Muriate of Cocaine in the Dental Infirmary of the University of Maryland. By Chas. L. Steel, D. D. S., M. D. Demonstrator of Operative Dentistry .... 475

### MONTHLY SUMMARY.

Dakota Dental Law .... 477

